# Program Implementation and Church Members’ Health Behaviors in a Countywide Study of the Faith, Activity, and Nutrition Program

**DOI:** 10.5888/pcd18.200224

**Published:** 2021-01-14

**Authors:** John A. Bernhart, Sara Wilcox, Ruth P. Saunders, Brent Hutto, Jessica Stucker

**Affiliations:** 1 Prevention Research Center, Arnold School of Public Health, University of South Carolina, Columbia, South Carolina; 2 Department of Health Promotion, Education, and Behavior, Arnold School of Public Health, University of South Carolina, Columbia, South Carolina; 3 Department of Exercise Science, Arnold School of Public Health, University of South Carolina, Columbia, South Carolina

## Abstract

Implementation research of health programs in faith-based organizations is lacking. The Faith, Activity, and Nutrition (FAN) program helps churches improve physical activity and fruit and vegetable behaviors of members. This study examined associations between implementation of FAN intervention components and church members’ physical activity, fruit and vegetable behaviors, and self-efficacy for improving these behaviors. FAN was implemented in 35 churches in a southeastern US county. After attending in-person training, led by community health advisors, church committees received 12 months of telephone-delivered technical assistance to implement FAN according to 4 components: increasing opportunities, increasing guidelines and policies, increasing pastor support, and increasing messages for physical activity and healthy eating in their church. In this correlational study, FAN coordinators (n = 35) for each church reported baseline practices in 2015 and 12-month follow-up implementation of the 4 components for physical activity and healthy eating in 2016. Church members (n = 893) reported perceived implementation, physical activity and fruit and vegetable behaviors, and self-efficacy at 12-month follow-up in 2016. Independent variables were coordinator-reported baseline practices, baseline-adjusted 12-month implementation, and member-perceived 12-month implementation. Multilevel modeling examined associations between independent variables and member-reported 12-month physical activity and fruit and vegetable behaviors and self-efficacy. Coordinator-reported 12-month implementation of fruit and vegetable opportunities was associated with member fruit and vegetable consumption. Member perceptions at 12 months of church physical activity opportunities, pastor support, and messages were associated with higher self-efficacy for physical activity; pastor support and messages were positively associated with physical activity. Member perceptions at 12 months of fruit and vegetable opportunities, pastor support, and messages were associated with higher fruit and vegetable consumption and self-efficacy. Member-perceived implementation was more strongly associated with member behaviors than coordinator-reported implementation. Providing opportunities for healthy eating during already scheduled events may be an effective strategy for improving fruit and vegetable behavior.

**Summary**

**What is already known on this topic?**

Effectiveness studies have shown that faith-based programs lead to improved health behaviors.

**What is added by this report?**

This study reports the relationships between implementation components of a faith-based program and study outcomes from perspectives of program leaders and program members. Leader reports of opportunities predicted fruit and vegetable and physical activity behaviors among members. Member perceptions of opportunities, pastor support, and messages were related to their physical activity and fruit and vegetable intake behaviors and self-efficacy.

**What are the implications for public health practice?**

Faith-based programs should emphasize increasing opportunities for fruits and vegetables and physical activity to improve member health. Member perceptions of opportunities, pastor support, and messages may be important program targets.

## Introduction

The prevalence of many chronic diseases is higher among African Americans than among other racial/ethnic groups (1,2), and people living in rural areas have poorer health compared with people living in urban areas (3,4). Health programs that result from academic and faith-based partnerships may help engage these priority populations and contribute to health equity (5). For example, previous faith-based programs have focused on supporting pastors and/or other lay health leaders (6) and addressing other leverage points of health behavior change, including behavior modification (7), proposing policy, and changing the church environment (8).

Research to date has primarily focused on assessing the effectiveness of these academic- and faith-based programs. Systematic reviews conducted by Bopp and colleagues (9) and Tristão Parra and colleagues (10) concluded that faith-based interventions increased physical activity behaviors. In addition, a systematic review by Lancaster and colleagues (11) identified a high success rate of faith-based programs designed to reduce weight and improve eating behaviors. Despite positive findings in these systematic reviews, few studies identified in the reviews focused on organizational changes within the church. Furthermore, little research exists that examines associations between implementation of specific intervention components and outcomes, warranting the need for implementation research of these evidence-based programs.

To understand implementation of faith-based programs, researchers must plan, a priori, a sound study design to measure the implementation of an intervention’s components. Furthermore, it is important to assess the relationship between implementation of specific intervention components and study outcomes (12). This type of assessment informs future design of programs, thereby leading to enhanced translation of evidence-based programs into communities and other settings (13).

The Faith, Activity, and Nutrition (FAN) program had improved physical activity and fruit and vegetable behaviors among church members in 2 group-randomized trials (8,14). The FAN intervention is a faith-based (vs faith-placed) intervention that incorporates spirituality and focuses on emic changes within the church (5). FAN is based on an ecologic model (15) in which church committees were trained to implement the 4 FAN components in their churches: 1) increase physical activity and healthy eating opportunities before, during, or after already scheduled events, 2) create supportive physical activity and healthy eating policies (guidelines), 3) enlist the support of the pastor, and 4) share physical activity and healthy eating messages (8). In the FAN dissemination and implementation study, research staff trained community health advisors (16) to deliver the training and technical assistance to church committees.

In the first phase of the FAN dissemination and implementation study (8), a county-wide initiative that used a group randomized design, church coordinators (ie, FAN coordinators) in early intervention churches reported significantly higher levels, compared with delayed intervention control churches, of overall implementation for physical activity components and most healthy eating components (17). Although Saunders and colleagues reported significant increases in the overall implementation of most physical activity and healthy eating components, they did not examine the associations between implementation of each component and member physical activity and fruit and vegetable behaviors and self-efficacy. Increasing our understanding of these relationships may shed light on which intervention components relate to which outcomes, thereby informing future scale-up and dissemination of the program.

## Purpose and Objectives

The purpose of this study was to expand previously reported FAN implementation findings (17) by examining associations between implementation of intervention components for physical activity and healthy eating (ie, opportunities, guidelines, pastor support, and messages) and member behaviors and self-efficacy. First, we examined associations between FAN coordinator–reported practices at baseline and physical activity and fruit and vegetable behaviors and self-efficacy among church members at 12 months. Second, we examined associations between FAN coordinator–reported 12-month implementation of intervention components (adjusted for baseline practices) and church members’ physical activity and fruit and vegetable behaviors and self-efficacy at 12 months. Lastly, we examined associations between church members’ perceived implementation of intervention components and members’ physical activity and fruit and vegetable behaviors and self-efficacy. Although the healthy eating component of FAN emphasizes multiple aspects of positive nutrition behaviors (increased intake of fruit, vegetables, and whole grains and decreased intake of saturated fat and sodium), we assessed only fruit and vegetable consumption and self-efficacy. We hypothesized that higher levels of FAN coordinator– and member-perceived implementation of the FAN intervention components at 12 months would be associated with higher levels of physical activity and fruit and vegetable behaviors and self-efficacy among church members.

## Intervention Approach

Data for this study come from a larger dissemination and implementation study, described elsewhere (8). In brief, committees (3–5 church members) led by a FAN coordinator attended an in-person training led by community health advisors (16). During training, the community health advisors guided church leaders and committees through an interactive assessment and planning process to develop a plan for implementing 4 organizational and environmental components for physical activity and healthy eating in the church based on Cohen’s structural model of health behaviors (15). Except for a few core program activities, church committees had freedom to choose activities tailored to the needs, interests, and culture of their church. After training, church committees completed and submitted a FAN program plan that detailed program implementation for the next 12 months. The community health advisors conducted brief monthly technical assistance calls (1 per month) with FAN coordinators or pastors to support implementation.

In the first phase of the FAN dissemination and implementation study, all churches (n = 132) in the selected county were invited to participate. Fifty-nine churches agreed to be randomized to an early (n = 39) or delayed (n = 20) intervention group. Churches in the early intervention group attended FAN training in 2015, and churches in the delayed intervention control group attended FAN training in 2016. In total, 54 churches attended training and completed baseline and follow-up evaluations and surveys. Churches were most commonly of Baptist denomination (45%) or nondenominational or independent (20%), identified as predominantly African American congregations (97%), and had 25 to 49 members (36%) or 50 to 74 members (25%). We limited analyses to early intervention churches (n = 35) where we had baseline and 12-month data from FAN coordinators and 12-month data from members. The purpose of this study was to examine associations between FAN component implementation and member-reported outcomes. Thus, we only included churches where training was conducted on program implementation. Member surveys were not repeated 12 months after the delayed intervention churches received training. Results comparing early intervention with delayed intervention churches at 12 months are reported elsewhere (8,17).

## Evaluation Methods

### Study design

Before attending training, staff members at the Survey Research Laboratory at the University of South Carolina conducted baseline interviews with FAN coordinators, where they reported their church’s baseline practices of the 4 FAN intervention components during the previous year. Twelve months later, interviews were conducted with FAN coordinators, who reported their church’s implementation of the same FAN intervention components during the previous year.

Church members’ physical activity and fruit and vegetable behaviors, self-efficacy for physical activity and fruit and vegetable behaviors, and perceived implementation of opportunities, pastor support, and messages were assessed only at 12 months, not at baseline. The collection of these data at baseline was not feasible (visiting each church was time consuming and costly), and the randomized design increased the likelihood of baseline equivalence across groups.

### Data collection

The Survey Research Laboratory completed baseline interviews with 39 FAN coordinators from early intervention churches from September 2, 2015, through October 28, 2015, (100% response rate) and completed 12-month implementation interviews with 35 FAN coordinators from September 6, 2016, through November 3, 2016 (97% of trained churches; 90% of randomized churches). We found only 1 instance in which the FAN coordinator who completed the baseline interview was different from the FAN coordinator who completed the 12-month implementation interview.

Trained data collectors visited churches during summer 2016 to administer surveys to church attendees and complete environmental audits (8,18,19). Attendees were eligible to complete the survey if they were aged 18 or older and attended services at the church at least once per month. In total, 893 surveys were completed by attendees from 35 early intervention churches; we did not include surveys from control churches in this analysis.

Characteristics of the full sample (ie, intervention and delayed intervention) of church members are reported elsewhere (8).

### Measures

**Baseline practices and 12-month implementation.** The measures for baseline practice and 12-month implementation were adapted from the previous FAN study (14,17,20) and were based on the underlying ecologic model. Additional review from the FAN Dissemination and Implementation Evaluation Committee ensured consistency of survey items with the conceptual model (15). Baseline practices and 12-month implementation of each of the 4 FAN components for physical activity and healthy eating were reported by the FAN coordinator from each church. Baseline practices were assessed in 2015, and 12-month implementation was assessed in 2016. In total, physical activity components were assessed with 11 items: 4 for opportunities, 1 for guidelines/policies, 2 for pastor support, and 4 for messages. Healthy eating components were assessed with 9 items: 2 for opportunities (focused only on fruits and vegetables), 2 for guidelines/policies (focused only on fruits and vegetables), 1 for pastor support, and 4 for messages. All items were assessed by using a 4-point frequency Likert scale where a 1 represented “rarely or never” or “not at all,” 2 represented “very little or every few months,” 3 represented “some of the time or about monthly,” and 4 represented “about weekly” or “almost all of the time.” When components were assessed with multiple questions, an average rating was calculated for that component. Scores for each component could range from 1 to 4.

**Perceived 12-month implementation.** Church members reported perceived implementation at follow-up only in 2016 on the same 4-point frequency Likert scale (21). In total, physical activity components were assessed with 11 items: 4 for opportunities, 2 for pastor support, and 5 for messages. Healthy eating components were assessed with 7 items: 2 for fruit and vegetable opportunities, 1 for pastor support, and 4 for messages. An average rating was calculated for each component. Similar to measures assessing baseline practices, scores for each component could range from 1 to 4.

**Physical activity.** Physical activity was measured at the 12-month follow-up by using the 6-item physical activity module from the 2009 Behavioral Risk Factor Surveillance System (22). Although newer modules were available, we selected this module to remain consistent with our measures of the FAN program over time, because the 2009 module was shorter and easier to administer by telephone or via online surveys, and because the 2009 module was shown to have group-level validity (22,23). Participants reported (ie, yes/no) whether they did at least 10 minutes of moderate activities (examples provided) in a usual week, and if so, days per week and total time per day. These same items were repeated for vigorous activities. Members were categorized as inactive if they answered no to doing moderate- or vigorous-intensity activities for at least 10 minutes at a time. They were classified as meeting the 2008 Physical Activity Guidelines (24) (in place when the study was conducted) if they reported at least 150 minutes per week of moderate- or vigorous-intensity physical activity (vigorous minutes were multiplied by 2 and added to moderate minutes).

**Fruit and vegetable consumption.** Fruit and vegetable consumption was assessed at the 12-month follow-up only. We provided a list of examples of 1-cup equivalents of fruits and vegetables. After providing the list, we asked 2 questions: “About how many cups of fruit (including 100% pure fruit juice) do you eat or drink each day?” and “About how many cups of vegetables (including 100% pure vegetable juice) do you eat or drink each day?” The response options were open-ended for participants to fill in the number of cups per day. This measure was used previously in a faith-based study (25–27).

**Self-efficacy.** Self-efficacy was assessed at the 12-month follow-up only among church members. Members answered 5 items for overcoming barriers to physical activity from 1 (not at all confident) to 7 (very confident) (28) and 8 items for overcoming barriers to fruit and vegetable consumption (25). Items were averaged within each scale, and each scale could range from 1 to 7. Internal consistency for physical activity self-efficacy was α = 0.88 and fruit and vegetable self-efficacy was α = 0.92.

### Data analysis

For our analyses, the dependent variables consisted of church members’ physical activity (ie, inactive or meeting physical activity guidelines), fruit and vegetable behaviors (ie, cups of fruits and vegetables consumed per day), and self-efficacy for physical activity and consuming fruits and vegetables.

To account for the high correlation between FAN coordinator reports of baseline practices and 12-month implementation of corresponding components, we created a residualized change score for each physical activity and healthy eating component at 12 months. By doing so, we were able to use the baseline practice score and the residualized 12-month change score simultaneously in a single model. Otherwise, the collinearity of baseline practices and unadjusted 12-month implementation would require each component to be modeled separately. Effect sizes for each component were calculated by subtracting the baseline score from the 12-month score and dividing by the baseline standard deviation and interpreted with guidelines from Cohen and colleagues for small (Cohen *d* = 0.2), medium (Cohen *d* = 0.5), and large (Cohen *d* = 0.8) effects (29).

We then conducted a series of multilevel models. The 5 distinct dependent variables were church members’ physical inactivity, meeting physical activity guidelines, physical activity self-efficacy, fruit and vegetable behaviors, and fruit and vegetable self-efficacy. We tested each FAN intervention component (opportunities, pastor support, guidelines/policies, and messages) individually in relation to the dependent variables. Physical activity components were tested relative to physical activity outcomes, and healthy eating components were tested relative to healthy eating outcomes. Thus, for each model, we regressed the outcome on the FAN coordinator–reported baseline practice and the corresponding residualized 12-month change score. For example, in a single model, we regressed the outcome of church member physical activity self-efficacy on FAN coordinator–reported baseline report of offering physical activity opportunities and the residualized 12-month change score of physical activity opportunities. We conducted similar models regressing church member outcomes on church members’ perceived implementation of each FAN component.

All models accounted for the clustering of members within churches. Missing data from members reduced the sample size for some of the models. SAS *proc mixed* was used for continuous outcomes of fruit and vegetable cups consumed per day and physical activity and fruit and vegetable self-efficacy. SAS *proc glimmix* was used for categorical outcomes of physical inactivity and meeting 2008 physical activity guidelines. Significance was determined a priori at the .05 level. All analyses were completed using SAS version 9.4 (SAS Institute Inc).

## Results

A total of 811 surveys were completed by members of the 35 early intervention churches (mean, 25.5 surveys per church; range, 5–77). In early intervention churches, church members were, on average, aged 53.0 (SD, 15.6), had an average body mass index (kg/m^2^) of 31.3 (SD, 6.9), were women (70%), and African American (95%) (Table 1). In addition, most of the sample had at least a high school education (90%). Most church members (71%) reported regularly meeting physical activity guidelines and few (11%) reported physical inactivity. The average fruit and vegetable consumption was 4.1 (SD, 2.3) cups per day, physical activity self-efficacy score was 3.8 (SD, 1.5) points, and fruit and vegetable self-efficacy score was 4.9 (SD, 1.4) points. FAN coordinators from early intervention churches were, on average, aged 59.7 (SD, 9.4), predominantly women (94%), and African American (97%). In addition, more than half (60%) of FAN coordinators had at least some college or technical school degree.

**Table 1:** Demographic Characteristics of Faith, Activity, and Nutrition (FAN) Coordinators and Church Members From Early Intervention Churches in the FAN Program, 2015^a^

Characteristic	FAN Coordinators (n = 35)	Church Members (n = 811)
**Age, mean (SD), y**	59.7 (9.4)	53.0 (15.6)
**Sex**		
Female	33 (94)	568 (70)
Male	2 (6)	243 (30)
**Education**		
<High school graduate	1 (3)	85 (10)
High school graduate	13 (37)	321 (40)
1–3 Years of college or technical school	11 (31)	220 (27)
College graduate	10 (29)	185 (23)
**Race/ethnicity**		
Black or African American	34 (97)	773 (95)
White or other	1 (3)	30 (4)
**Physical activity**		
Inactive	NA^b^	95 (11)
Meets physical activity guidelines	NA^b^	597 (71)
**Mean (SD) no. of fruit and vegetable cups per day**	NA^b^	4.1 (2.3)
**Physical activity self-efficacy, mean (SD)^c^**	NA^b^	3.8 (1.5)
**Fruit and vegetable self-efficacy, mean (SD)^d^**	NA^b^	4.9 (1.4)

Abbreviation: NA, not assessed.

a Data were collected from surveys administered to FAN coordinators and church members in 2015. Values are number (percentage) unless otherwise indicated; percentages may not add to 100 because of rounding.

b Not assessed among FAN coordinators.

c Church members answered 5 items for overcoming barriers to physical activity from 1 (not at all confident) to 7 (very confident) (28).

d Church members answered 8 items for overcoming barriers to fruit and vegetable consumption from 1 (not at all confident) to 7 (very confident) (25).

### Implementation

FAN coordinators reported higher levels of implementing intervention components at 12 months than at baseline (Table 2). For example, implementation of mean physical activity opportunities at 12 months was rated 2.7 (SD, 0.5) compared with 1.9 (SD, 0.7) at baseline. Implementation of fruit and vegetable opportunities was rated 3.6 (SD, 0.6) at 12 months and 3.4 (SD, 0.5) at baseline. Members and FAN coordinators reported similar implementation levels at 12 months. For example, member-perceived 12-month implementation of physical activity opportunities was rated 2.7 (SD, 0.8) and FAN coordinators–perceived implementation was rated 2.7 (SD, 0.5). Similarly, member-perceived 12-month implementation of fruit and vegetable opportunities was rated 3.4 (SD, 0.6) and FAN coordinators–perceived implementation was rated 3.6 (SD, 0.6).

**Table 2:** Faith, Activity, and Nutrition (FAN) Coordinator Reports of Baseline Practices and 12-Month Implementation Components, and Member-Perceived Implementation at 12 Months, 2015–2016^a^

Component of FAN Implementation	FAN Coordinator–Reported Practice at Baseline, Mean (SD) Score	FAN Coordinator–Reported Practice Implementation at 12 Months, Mean (SD) Score	FAN Coordinator Effect Size, Cohen *d* b	Church Member–Perceived Implementation at 12 Months, Mean (SD)
**Physical activity**				
Opportunities	1.9 (0.7)	2.7 (0.5)	1.2	2.7 (0.8)
Guidelines	2.0 (0.7)	2.8 (1.1)	1.3	NA
Pastor support	1.3 (0.5)	2.4 (0.7)	2.5	2.4 (0.9)
Messages	1.6 (0.7)	2.7 (0.5)	1.5	2.7 (0.8)
**Fruits and vegetables**				
Opportunities	3.4 (0.5)	3.6 (0.6)	0.4	3.4 (0.6)
Guidelines	2.3 (0.8)	3.2 (1.0)	1.2	NA
**Healthy eating**				
Pastor support	1.8 (0.8)	2.9 (0.7)	1.4	2.9 (0.9)
Messages	1.9 (0.8)	2.9 (0.6)	1.4	3.0 (0.8)

Abbreviation: NA, not assessed.

a All items were assessed by using a 4-point frequency Likert scale where a 1 represented “rarely or never” or “not at all,” 2 represented “very little or every few months,” 3 represented “some of the time or about monthly,” and 4 represented “about weekly” or “almost all of the time.”

b Cohen *d* calculated as (12-month implementation score − baseline practice score)/baseline SD.

### FAN coordinator–reported implementation and church member behaviors

Providing more physical activity opportunities at baseline was significantly associated with church members meeting physical activity guidelines at the 12-month follow-up (odds ratio [OR] = 1.38, 95% CI, 1.03–1.83) (Table 3). In addition, residualized 12-month change in fruit and vegetable opportunities was significantly associated (β = 0.46 [SE, 0.21]; *F* = 4.54; *P* = .04) with fruit and vegetable consumption among church members at 12-month follow up. No other baseline or residualized 12-month change components were significantly associated with the behaviors or self-efficacy of church members.

**Table 3:** Faith, Activity, and Nutrition (FAN) Coordinator–Reported Baseline Practices and Baseline-Adjusted 12-Month Change Scores in Implementation Components on Church Member Physical Activity, Fruit and Vegetable Intake, and Self-Efficacy^a^

Independent Variable^b^	Dependent Variables							
	Physical Inactivity, OR (95% CI)	Meeting Physical Activity Guidelines, OR (95% CI)	Physical Activity Self-Efficacy		Cups per Day of Fruits and Vegetables		Fruit and Vegetable Self-Efficacy	
			β (SE)	*F* (*P* Value)	β (SE)	*F* (*P* Value)	β (SE)	*F* (*P* Value)
**Opportunities models**								
Baseline physical activity and fruit and vegetable opportunities	0.66 (0.42–1.03)	1.38 (1.03–1.83)	0.10 (0.09)	1.37 (.25)	0.25 (0.17)	2.29 (.14)	−0.02 (0.11)	0.05 (.83)
Residualized 12-month physical activity and fruit and vegetable change in opportunities	1.29 (0.74–2.25)	0.84 (0.59–1.21)	−0.15 (0.11)	1.79 (.19)	0.46 (0.21)	4.54 (.04)	0.09 (0.14)	0.46 (.50)
**Guidelines models**								
Baseline physical activity and fruit and vegetable guidelines	0.98 (0.61–1.59)	1.06 (0.78–1.45)	0.12 (0.09)	1.75 (.19)	0 (0.12)	0 (.98)	0.10 (0.06)	2.53 (.11)
Residualized 12-month physical activity and fruit and vegetable change in guidelines	1.08 (0.76–1.53)	1.06 (0.85–1.33)	−0.03 (0.06)	0.20 (.66)	−0.10 (0.10)	1.02 (.33)	−0.06 (0.05)	1.28 (.26)
**Pastor support models**								
Baseline pastor support for physical activity or healthy eating	1.07 (0.59–1.94)	1.01 (0.67–1.51)	0.13 (0.12)	1.24 (.27)	−0.02 (0.13)	0.04 (.84)	0.04 (0.07)	0.30 (.59)
Residualized 12-month change in pastor support for physical activity or healthy eating	1.08 (0.67–1.75)	1.08 (0.79–1.49)	0.15 (0.09)	2.84 (.10)	0.07 (0.14)	0.25 (.62)	0 (0.08)	0 (.98)
**Messages models**								
Baseline physical activity or healthy eating messages	0.98 (0.64–1.50)	1.06 (0.80–1.41)	0.06 (0.08)	0.48 (.50)	0.06 (0.12)	0.24 (.63)	0.07 (0.07)	0.83 (.37)
Residualized 12-month change in physical activity or healthy eating messages	0.91 (0.47–1.74)	1.09 (0.71–1.67)	−0.07 (0.13)	0.26 (.62)	−0.04 (0.18)	0.05 (.82)	−0.01 (0.11)	0 (.95)

Abbreviation: OR, odds ratio.

a Each model (20 models in total were tested) included baseline practices and baseline-adjusted 12-month implementation and accounted for member clustering within churches (SAS *proc mixed* for continuous outcomes; SAS *proc glimmix* for categorical outcomes).

b For physical activity outcomes, physical activity practices and implementation scores were used; for fruits and vegetables outcomes, healthy eating and fruits and vegetables practices and implementation scores were used.

### Church member-perceived implementation and church member behaviors

Members who perceived more physical activity opportunities (*F* = 17.78; *P* < .001), greater pastor support for physical activity (*F* = 15.95; *P* < .001), and more physical activity messages (*F* = 17.52; *P* < .001) in their church reported significantly higher physical activity self-efficacy (Table 4). Members who perceived greater pastor support for physical activity and more physical activity messages were less likely to be inactive (OR = 0.73, 95% CI, 0.56–0.95; OR = 0.66, 95% CI, 0.50–0.87, respectively) and more likely to meet physical activity guidelines (OR = 1.30, 95% CI, 1.09–1.54; OR = 1.30, 95% CI, 1.07–1.57). Members who perceived more fruit and vegetable opportunities (*F* = 53.42; *P* < .001), greater pastor support for healthy eating (*F* = 20.08; *P* < .001), and more healthy eating messages (*F* = 20.84; *P* < .001) reported significantly higher fruit and vegetable self-efficacy. In addition, members who perceived more fruit and vegetable opportunities (*F* = 28.22; *P* < .001), greater pastor support for healthy eating (*F* = 5.91; *P* = .02), and more healthy eating messages (*F* = 5.96; *P* = .02) reported significantly higher daily consumption of fruits and vegetables.

**Table 4:** Associations Between Church Member–Perceived Implementation and Church Member Physical Activity, Fruit and Vegetable Intake, and Self-Efficacy^a^

Independent Variable^b^	Dependent Variable							
	Physical Inactivity, Odds Ratio (95% CI)	Meeting Physical Activity Guidelines, Odds Ratio (95% CI)	Physical Activity Self-Efficacy		Cups per Day of Fruits and Vegetables		Fruits and Vegetables Self-Efficacy	
			β (SE)	*F* (*P* Value)	β (SE)	*F* (*P* Value)	β (SE)	*F* (*P* Value)
**Opportunities models**								
Perceived 12-month opportunities for physical activity or fruit and vegetable consumption	0.75 (0.56–1.01)	1.20 (0.98–1.48)	0.29 (0.07)	17.78 (<.001)	0.71 (0.13)	28.22 (<.001)	0.60 (0.28)	53.42 (<.001)
**Pastor support models**								
Perceived 12-month pastor support for physical activity or healthy eating	0.73 (0.56–0.95)	1.30 (1.09–1.54)	0.23 (0.06)	15.95 (<.001)	0.22 (0.09)	5.91 (.02)	0.25 (0.06)	20.08 (<.001)
**Messages models**								
Perceived 12-month messages on physical activity or healthy eating	0.66 (0.50–0.87)	1.30 (1.07–1.57)	0.27 (0.06)	17.52 (<.001)	0.25 (0.10)	5.96 (.02)	0.28 (0.06)	20.84 (<.001)

a Each model (15 models were tested) included member-perceived 12-month implementation and accounted for member clustering within churches (SAS *proc mixed* for continuous outcomes; SAS *proc glimmix* for categorical outcomes). The implementation components of physical activity and fruit and vegetable guidelines were not assessed by members.

b For physical activity outcomes, physical activity practices and implementation scores were used; for fruit and vegetable outcomes, healthy eating and fruits and vegetables practices and implementation scores were used.

## Implications for Public Health

Assessing relationships between implementation of intervention components and outcomes increases the ability to translate known components for improving outcomes in future evidence-based programs (13). This study examined relationships between the implementation of the 4 FAN components (ie, opportunities, guidelines, pastor support, and messages) and church member physical activity and fruit and vegetable behaviors and self-efficacy.

### Implementation

This study confirms and expands previous research from Saunders and colleagues’ assessment of overall changes in implementation (17). FAN coordinator reports of 12-month implementation of each of the components increased from baseline practices. Second, FAN coordinator reports of 12-month implementation closely mirrored member perceptions of 12-month implementation. This consistent finding from 2 categories of respondents increases our confidence that our characterization of the FAN implementation environment is accurate. Overall, the change from baseline practice to 12-month implementation reflected in FAN coordinator reports was large for all components except fruit and vegetable opportunities (which was relatively high at baseline), which increased moderately. Collectively, these findings provide further support for evidence of FAN’s effectiveness in creating environmental changes in church settings.

### FAN coordinator–reported implementation and church member behaviors

Higher levels of FAN coordinator–reported baseline practices of physical activity opportunities were associated with church members meeting physical activity guidelines at 12 months, although residualized 12-month change scores in implementation were not. Only one of our findings in FAN coordinator–reported models aligned with our hypotheses. Higher levels of FAN coordinator–reported 12-month implementation of fruit and vegetable opportunities were significantly associated with members’ fruit and vegetable consumption at 12 months. For fruit and vegetable behaviors, providing opportunities may be the most important FAN intervention component for supporting these behaviors. Because churches often provide food at events, these regularly scheduled occurrences may have afforded churches in our study a simpler method to increase serving healthy foods to members. No other healthy eating intervention components, as reported by FAN coordinators, were significantly associated with fruit and vegetable behaviors or self-efficacy. It should be noted that although FAN emphasized multiple aspects of healthy eating (increased intake of fruits and vegetables and whole grains and decreased intake of saturated fat and sodium), we only assessed fruit and vegetable consumption and self-efficacy.

### Perceived church member implementation and church member behaviors

As hypothesized, members who perceived higher levels of physical activity opportunities, pastor support, and messages at 12 months also reported higher physical activity self-efficacy. In addition, members’ perceptions of greater pastor support and messages, but not physical activity opportunities, related to member physical activity; both were associated with lower odds of physical inactivity and higher odds of meeting physical activity guidelines. Members’ perceptions of each healthy eating intervention component were positively associated with fruit and vegetable consumption and self-efficacy for fruit and vegetable consumption.

We observed fewer associations between FAN coordinator–reported implementation and member outcomes compared with church member–perceived implementation and outcomes. These differing relationships may be attributed in part to the smaller sample size of the FAN coordinators (n = 35) and the resulting reduced power for the FAN coordinator–reported models. The greater associations between member-reported implementation and member outcomes may also be an artifact of the mono-method bias due to members self-reporting their physical activity and fruit and vegetable behaviors, self-efficacy, and perceived implementation (30). In addition, unlike FAN coordinators who reported both baseline practices and 12-month implementation of the FAN components, members were only assessed at 12 months. Thus, we were unable to adjust for baseline differences in member-perceived implementation models.

### Limitations and strengths

This study had limitations. First, we did not include data from the delayed intervention control group churches. The goal of this study was to evaluate how the implementation of specific intervention components contributed to member outcomes in faith-based settings, not to assess the effectiveness of the FAN intervention, as has been previously done (8,14). Second, because of feasibility and costs, we did not collect baseline measures among members, limiting our ability to assess changes in behaviors with changes in implementation. However, the randomization was effective, because the churches were generally well matched at baseline (8). In this dissemination and implementation study, the differences between the control and intervention group at 12 months (8,17) were consistent with earlier FAN effectiveness studies that also used a randomized design with before-and-after measurements of members (14,31). The direction and magnitude of the results replicated the results of previous studies, strengthening our confidence that the FAN intervention is responsible for changes in church-level implementation. Third, the small sample size of churches potentially limited our ability to detect associations between FAN coordinator reports of implementation and member behaviors, even though the sample size of church members was larger. Fourth, the member-reported associations of 12-month implementation and fruit and vegetable and physical activity behaviors and self-efficacy were subject to mono-method bias. Fifth, because of the nature of the items, we were unable to compute internal consistency (ie, Cronbach α) of our measures for baseline practice and implementation. For example, we did not expect to find agreement between the 2 items measuring fruit and vegetable opportunities and the 5 items measuring physical activity messages because these are distinct constructs. However, our measures were systematically developed through multiple trials (14,20) and expert review. Lastly, this study was limited to churches participating in FAN in a southern, rural, and medically underserved county. Thus, findings may have limited generalizability to churches in other areas of the United States.

Despite these limitations, our study also had strengths. First, it builds on previous implementation research of an evidence-based program (8,17). By examining associations between intervention components and outcomes, we can continue to improve and strengthen technical assistance for each component. Although not every component was associated with each of the study outcomes, FAN evaluation continues, and it is possible the implementation of the program as a whole contributed to improved health (8,17). Second, our study used an ecologic approach and measured associations between the environmental-level changes in the church (ie, implementation of intervention components) and individual-level behaviors and self-efficacy. We found that increased opportunities for fruit and vegetable consumption in the church setting related to increased fruit and vegetable consumption among members. In addition, programs like FAN that are guided by an ecologic approach reach more people than programs aimed at individual-level changes. Considering the large number of churches in rural, urban, and suburban areas, FAN is well-positioned to fit churches in any context, thereby improving health of communities. A third strength of this study is that we assessed environmental-level changes from 2 perspectives (ie, FAN coordinators and church members), leading to a fuller understanding of implementation in the church. Another strength of this study is the pre–post design for FAN coordinator–reported implementation, which enabled us to adjust for baseline practices congruent with the 4 FAN intervention components. Also, this study’s in-depth analysis of how each intervention component related to member outcomes provides researchers and practitioners with insights about the importance of offering opportunities to help church members engage in healthy behaviors (13). Lastly, church leaders and members were predominantly African American, demonstrating the relevance of partnering with faith-based organizations to reach high-priority populations and promote healthy living to decrease health disparities (5). In the African American community, faith-based interventions have been effective at improving health (32,33). Faith plays an important role in African American culture, and faith-based interventions such as FAN bring personal faith and church doctrine together. Because many churches often have health promotion efforts in place, faith-based interventions can support and expand existing health ministries to improve health (34).

The FAN program is an effective intervention for improving the health of church members in diverse settings, positioning it well for greater dissemination and implementation (8,14). Our study expanded on previous FAN implementation research (17) and explored how the implementation of specific intervention components was associated with church members’ behaviors and self-efficacy. We found that implementation of opportunities, as reported by FAN coordinators and church members, appeared most important for improving fruit and vegetable behaviors. Member-perceived implementation of most components of FAN related to member physical activity and fruit and vegetable behavior and self-efficacy. Future studies should examine member-perceived practices and behaviors preintervention in addition to postintervention and emphasize increasing fruit and vegetable opportunities to help members improve fruit and vegetable behaviors.
